# Bacterial Colony from Two-Dimensional Division to Three-Dimensional Development

**DOI:** 10.1371/journal.pone.0048098

**Published:** 2012-11-14

**Authors:** Pin-Tzu Su, Chih-Tang Liao, Jiunn-Ren Roan, Shao-Hung Wang, Arthur Chiou, Wan-Jr Syu

**Affiliations:** 1 Institute of Microbiology and Immunology, National Yang-Ming University, Taipei, Taiwan, Republic of China; 2 Department of Physics, National Chung-Hsin University, Taichung, Taiwan, Republic of China; 3 Center of Biophotonics, National Yang-Ming University, Taipei, Taiwan, Republic of China; 4 Department of Microbiology, Immunology and Biopharmaceuticals, National Chiayi University, Chiayi, Taiwan, Republic of China; Loyola University Medical Center, United States of America

## Abstract

On agar surface, bacterial daughter cells form a 4-cell array after the first two rounds of division, and this phenomenon has been previously attributed to a balancing of interactions among the daughter bacteria and the underneath agar. We studied further the organization and development of colony after additional generations. By confocal laser scanning microscopy and real-time imaging, we observed that bacterial cells were able to self-organize and resulted in a near circular micro-colony consisting of monolayer cells. After continuous dividing, bacteria transited from two-dimensional expansion into three-dimensional growth and formed two to multi-layers in the center but retained a monolayer in the outer ring of the circular colony. The transverse width of this outer ring appeared to be approximately constant once the micro-colony reached a certain age. This observation supports the notion that balanced interplays of the forces involved lead to a gross morphology as the bacteria divide into offspring on agar surface. In this case, the result is due to a balance between the expansion force of the dividing bacteria, the non-covalent force among bacterial offspring and that between bacteria and substratum.

## Introduction

In the process of continuous division of bacteria, interactions among bacterial offspring and that between bacteria and surroundings may result in a developing bacterial micro-colony [1], [2]. These interactions could range from any loose contact to an intimate adhesion. After initiation of adhesion, bacteria may colonize and form aggregates of different patterns. This colonization process may further develop into complex organization termed biofilm [3]. Biofilms may confer bacterial resistance to many environmental conditions that are hostile to the bacteria. One of the examples is that in a flow cell culture system, cells of ampicillin-sensitive *E. coli* have generated subpopulations with ampicillin resistance in the deep layers of biofilms [4].

Another complication caused by a biofilm formation is that bacterial cells are confined to a limited space [5] and this kind of growth had been observed in *Listeria*, *Salmonella* and *Clostridium* [6], [7]. Bacterial cells within a restricted space may have to respond to an unfavorable surrounding during their expansion. Moreover, a new concept about “social intelligence” has been suggested to both bacterial colony and growing tumor as they are forming smart communities of cooperative cells [8]. Also, recent studies have revealed that bacterial cells self-organize into high-density colonies in a pattern perhaps similar to the collective motions commonly seen with fish schools and flocking birds [9], [10], [11]. The highly self-organized arrangement in bacterial colonies has been attributed to the enhancement of bacterial access toward nutrients and favorable evacuation of waste out of the colony interior [12]. Therefore, an intervention to prevent the formation of bacterial aggregate at the early phase (reviewed in [13], [14]) could be an effective way to prevent the formation of a tough biofilm.

To understand the formation of bacterial colonies, we have previously addressed the early events during the first two rounds of bacterial divisions [15], and here we tackle the issue on how the bacterial growth transit from a two-dimensional expansion to form a three-dimensional micro-colony that remains hard to see by naked eyes after 8 h of growth. To examine this, we grew enterohemorrhagic *Escherichia coli* (EHEC) O157:H7 on 1.5% LB-agar and followed the dynamics of growing cells through the formation of micro-colonies. We applied simultaneously both time-lapse imaging and confocal laser scanning microscopy (CLSM) [16] to investigate the sequential changes of the offspring organization. We observed that the bacterial population increased at the beginning to result in a single-cell layer arranged in a nearly circular, pie-like appearance. As the growth of bacteria and division continued, the diameter of the pie-like monolayer increased and a two-cell layer appears at the center of the bacterial lawn to form a center core that expanded outwards on the agar plane and upward in the third dimension as well. Under the microscope, the central zone of the micro-colony could be distinguished from the outermost single-cell ring; the later appeared to form dynamically at the edge of the micro-colony and was discernable from the central zone. We also noticed that the outermost single-cell ring reached a constant ring width after a period of growth.

## Materials and Methods

### Bacterial strains

EHEC O157:H7 strain 43888 from ATCC [17] was used and transformed with GFP-expressing plasmid derived from pQE60 (Qiagen).

### Bacterial culture and growth of micro-colony

Routinely, bacteria were cultured in Luria-Bertani broth (LB) and supplemented with or without 100 µg/ml ampicillin. To observe the micro-colony formation, overnight cultured bacteria were 1∶100 diluted in LB and grown until OD_600_ reached between 0.3–0.6. Bacteria were then appropriately diluted in LB and seeded on the top of thin LB-agar (1.5%; w/v) coated on a coverslip (18×18 mm) that was inverted and then assembled with a lower coverslip (22×22 mm). During the assembly, two spacers with 0.5-mm thickness were inserted between the two coverslips. As a result, a chamber was formed so that LB medium could be filled in at the beginning and occasionally added to prevent agar from drying. Bacterial cells on agar coverslips were observed with a Leica DM IRBE inverted microscope equipped with a 100×1.40 N.A. oil objective (Leica 506042) and an ORCA-ER digital camera (Hamamatsu C4742-95). Time-lapse images were taken with the assistance of MetaMorph program (Molecular Devices).

### Angular analysis of bacterial orientation in the outmost areas

To examine the bacterial orientation in the outermost areas of the bacterial micro-colonies, we developed a method for angular analysis. This method was modified from that previously used to study a highly ordered domain within swarming groups of *Myxococcus xanthus* [18]. In brief, a circle was drawn to fit the bacterial micro-colony and the center of the circle could be pinpointed since the bacterial micro-colonies observed under microscope are nearly circular. And then, an outermost ring with a ring-width of 2 µm (the length of a bacterium) was regarded as the outermost loop 1, and a 2^nd^ ring (again with a ring width of 2 µm) at the immediate inner neighbor was regarded as loop 2. To evaluate the bacterial orientation, double-headed arrows were drawn from end-to-end of individual cells. A line from the center of bacterial colony to the center of the double-headed arrow was then drawn; the acute angle formed between this line and the double-headed arrow (i.e. bacterial body) was then measured.

### CLSM image acquisition and analysis

To follow the third-dimensional growth of a micro-colony, bacteria growing on the agar-coated slides were covered with LB medium and stood still at 37°C in a temperature-controlled device, Chamlide TC (Live Cell Instrument, Seoul, Korea). Bacteria on the same focal plane were imaged with Leica SP5 confocal laser scanning microscope equipped with a 100×, 1.40 N.A. oil objective. A micro-colony was dissected from the top to the bottom, by optical sectioning with a thickness of either 0.5 µm or 0.05 µm, so that a three-dimensional image reconstruction could be facilitated. Reconstruction of the three-dimensional images of each micro-colony was carried out by using Imaris 6.3.1 software (Bitplane, Switzerland).

## Results

### Development of bacterial cells from a single cell to a colonial monolayer

**Figure 1:**
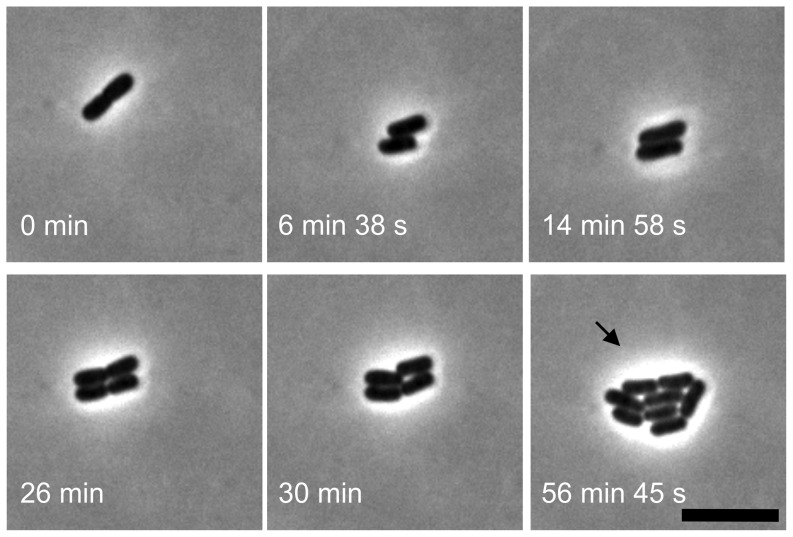
Observations of EHEC daughter cells' arrangements during the early division. EHEC O157:H7 was grown on a 1.5% LB-agar-coated slide (immersed in LB medium) and the early bacterial divisions were observed by time-lapsed microscopy. Daughter cells slid side-by-side after the first fission and then formed a 4-cell array after the 2^nd^ round of division. The arrow indicates the irregular arrangement of octomeric daughter cells that are to grow into a nearly circular micro-colony after a few more cycles of division. Scale bar: 5 µm.

**Figure 2:**
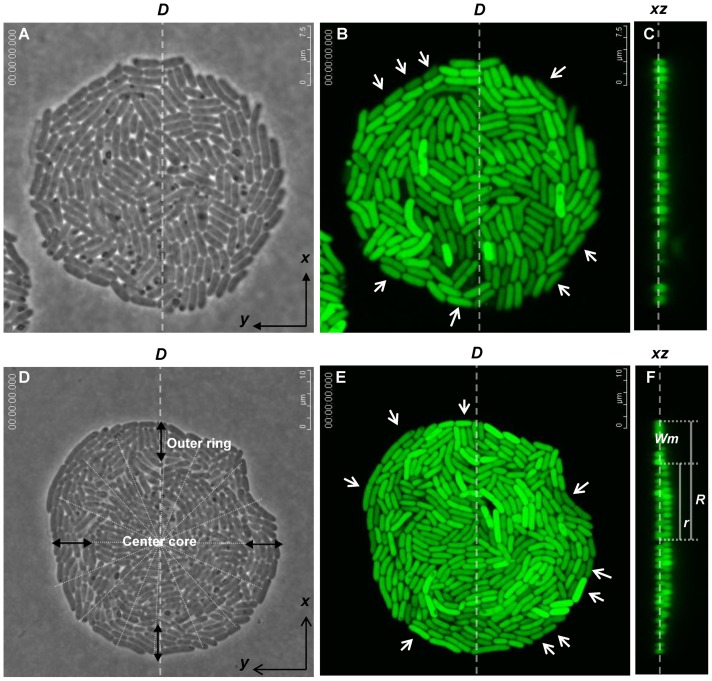
Micro-colonies observed by different microscopic imaging systems. GFP-expressing EHEC was cultivated as in Fig. 1. Micro-colony was observed: (A) on the *xy* plane with phase-contrast microscopy; (B) on the *xy* plane with CLSM; (C) on the *xz* plane along the diameter-line “*D*” with CLSM (sectioned at thickness of 0.5 µm). Images were taken after 4 h of cultivation. (D–F) Individual images were taken as that to (A–C), respectively, except for cultivation for 5 h. Note: a light outer ring and a dense center core concentrically seen in (D) are marked; the radius of the denser center core and that of the whole micro-colony are labeled “*r*” and “*R*”, respectively, in (F) and the difference between *r* and *R* is *W_m_*, the transverse width of a monolayer. Arrows indicate representative cells oriented approximately perpendicular to the radial direction of the micro-colony.

To follow the bacterial division, GFP-expressing EHEC O157:H7 was grown on slides coated with a thin layer of LB-agar (1.5%), which were subsequently immersed in LB medium cautiously. Images of the growing bacteria were taken with time-lapse recording by using both phase-contrast light microscopy and CLSM. Consistent with previous observations with non-pathogenic K-12 strain [1], [19], [20], the EHEC offspring slid side-by-side to each other and then formed a 4-cell array after the first two rounds of division. Irregular arrangements of daughter cells were observed after the third division (Fig. 1), and the micro-colony continued to develop in a fairly irregular random pattern. The bacteria were multiplying about every 0.5 h and grew in a form of a confluent monolayer on the LB agar. Figure 2A shows a micrograph of a micro-colony taken with the phase-contrast microscopy after 4 h of cultivation. Fig. 2B shows the image of GFP fluorescence emitted from the bacteria in the same micro-colony shown in Fig. 2A. Fig. 2B also reveals that the fluorescence intensities varied from cell to cell. At this stage, a sheet of bacterial monolayer was seen throughout this nearly circular micro-colony, as revealed by the fluorescence image shown in the *xz* cross-section (Fig. 2C), which was taken with CLSM along the diameter line “*D*” in Fig. 2B.

### Bacterial cells growing from monolayer to a structure with center of two layers

When observing the expansion of the offspring, a grey dot was seen first in the center of the nearly circular bacterial lawn and this dot gradually enlarged into a core as the micro-colony continued to develop. Fig. 2D shows a phase-contrast microscopic image of a micro-colony with a well-developed center core. When examined with a confocal microscope on the regular plane, the cell arrangement and GFP intensity variation from one bacterium to another (Fig. 2E) looked similar to those observed earlier as shown in Fig. 2B, except for an increasing bacterial number. However, the confocal laser-scanning microscopic image, the *xz* plane, revealed unambiguously that the micro-colony formed a distinct structure composing of a monolayer area at the outer ring and double layers at the center. We denote the radius of the central double layers by “*r*” and that of the whole bacterial lawn by “*R*”; hence the width “d” (or the diameter) of the central double layers is “*2r*” while the transverse width of the outer monolayer ring “*W_m_*” is “*R*-*r*” (Fig. 2F).

**Figure 3:**
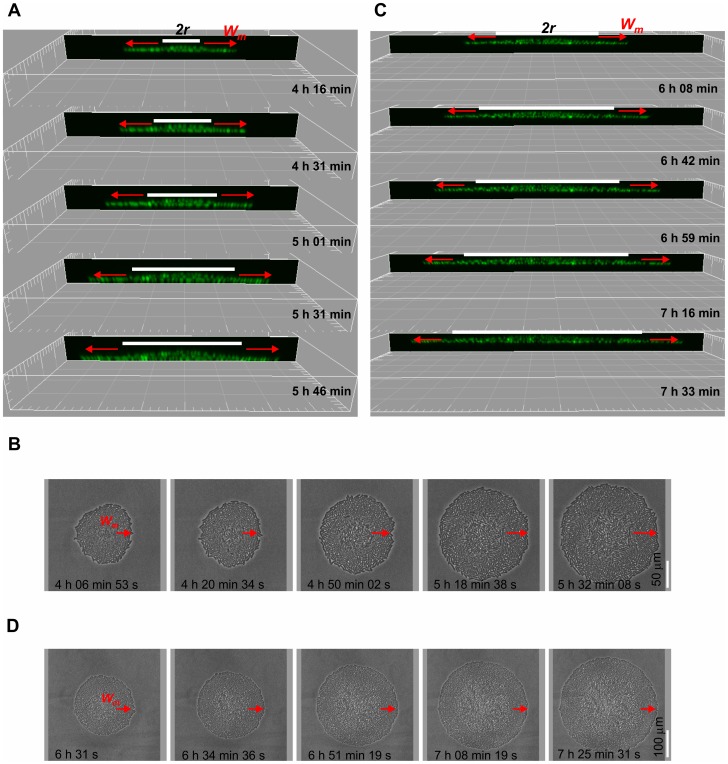
Time-lapse image analysis of the structures in the 3^rd^ dimension of a developing micro-colony. A growing bacterial micro-colony similar to that in Fig. 2 was followed by using a confocal laser scanning microscope (sectioned with a thickness of 0.05 µm). (A, C) Projected images at different time points for the *xz* plane after cross-sectioning along the *D* line of the micro-colony. *r*: the radius of the center core; *W_m_*, the transverse width of the outermost monolayer. (B, D) A series of images from the phase contrast microscopy. Arrows indicate the spaces of the monolayer rings. Scale of a gird in A and C: 10 µm.

To examine the micro-colony development closely, three-dimensional images of the colony in a time series were projected on the *xz* plane along a diagonal line. As shown in Fig. 3 (taken when cultivated for 4–7 h), the bacterial colony was expanding with enlarging gross diameter (*2R*). The central area of the bacterial colony, as defined by *2r* in Fig. 3A, extended gradually. Bacterial cells in this area were mostly stacked in two layers and a few cells near the very center started to be squeezed between the top and bottom layers (see image from 5 h 31 min and that from 5 h 46 min). When all outer rings where cells were seen with monolayer in Fig. 3A and Fig. 3B were examined, the ring-width *W_m_* seemed to remain the same, despite the increasing values of *both r and R*.

To investigate further whether *W_m_* reaches a constant while the center core of the micro-colony keeps expanding, bacterial growth was imaged up to 7 h 33 min, from the beginning of cultivation (Figs. 3C and 3D). Figure 3D shows the gross morphology of the colony, again, with the outer ring clearly noticeable under the phase-contrast microscope. The projected images on the *xz* plane (Fig. 3C) show that as the radius of the central area increased, the complexity of bacteria layers also increased, from two layers to multiple layers at the center of the core area. In contrast, the width of the monolayer area defined by ring-width *W_m_* appeared to be stable over the micro-colony expanding period.

### Measurement of the width of the monolayer cross-section of a micro-colony

At the time points of 5–6 h cultivation, the micro-colonies grew at sizes within the field of view of the phase-contrast microscope and micrographs of the entire colony could be captured. However, by 7 h, micro-colonies often grew to size beyond the field of view of the microscope. Also noticeable at this time, a center within the core area in the micro-colony gradually appeared and could be distinguishable by the phase contrast. From the *xz* plane projection, it is clear that the numbers of cell layers in the colony center cores increased progressively, from mostly 2–3 layers when cultivated for 5–6 h (Fig. 3A) and then to 2–4 layers after 7 h cultivation (Fig. 3C). In all cases, the middle of the central areas apparently had the largest number of cell layer.

**Figure 4:**
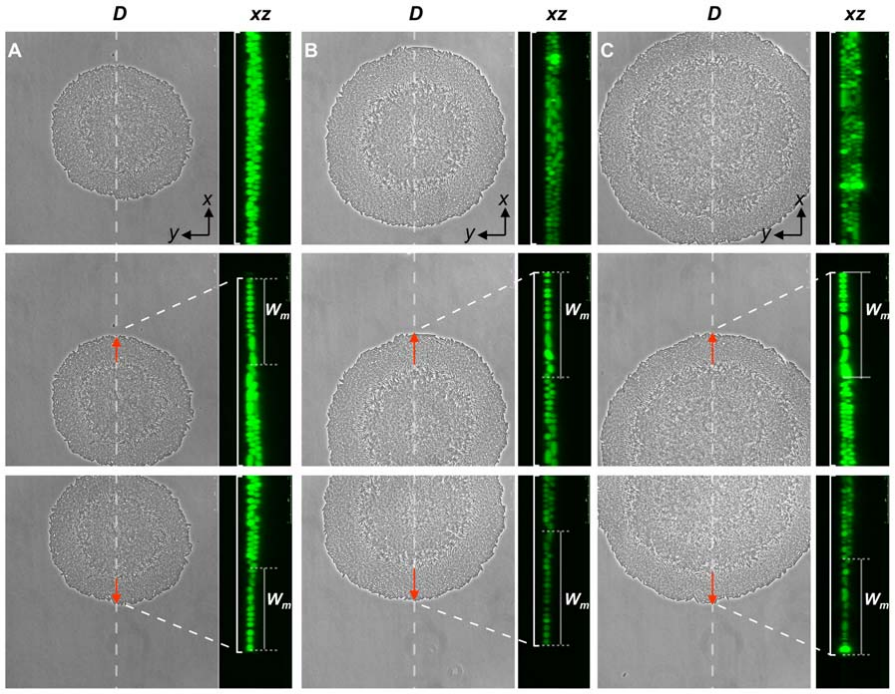
Imaging the multi-layer structure of micro-colony. Imaging was carried out as described earlier for the case of Fig. 2 that the sectioning thickness of CLSM was set at 0.5 µm. All phase-contrast images were taken at the same scale to show the change in sizes of growing micro-colonies: (A) 5 h; (B) 6 h; (C) 7 h. To the right of individual phase-contrast images are the *xz* plane along the *D* line of the micro-colony. In each vertical panel, the images were taken for the middle, upper and bottom parts of the micro-colony, respectively. Note: the magnification of the confocal florescence images were 3.5 times that of the phase contrast images.

**Figure 5:**
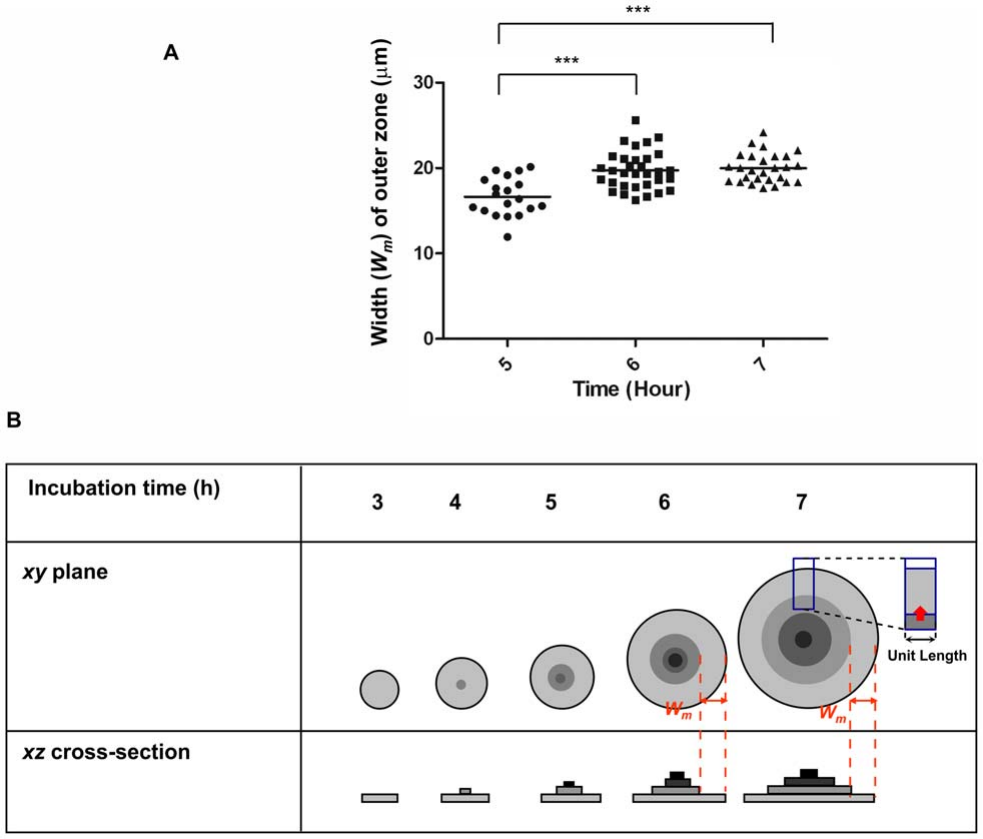
Characteristics summarized for the developing micro-colony. (**A**) **Measured widths of the outermost monolayer during micro-colony formation.** Measurements of *W_m_* values of individual micro-colonies were carried out similarly to that described in Fig. 5. Every single spot represents a mean of two *W_m_* values of a monolayer ring measured from images of the *xz* plane. Horizontal line marks the average of each measurement group. Three asterisked pairs indicate that there is a significant difference between the groups (*p*<0.0001 by *t*-test). (**B**) **Illustration of a micro-colony development by viewing at different planes.** Even though the diameter and layers of the bacterial micro-colony were increasingly expanding, the average width (*W_m_*) of the outermost monolayer reached a constant value after approximately 6 h of growth. The red arrow indicates that the constant outward force per unit length. Note: the boundaries and intensities between layers are not as sharp as illustrated, particularly those beyond the 2^nd^/3^rd^ layers, and scales are not proportionally represented.

To measure *W_m_* over a time period, sampled micro-colonies were cross-sectioned individually along line *D*. Figure 4 shows examples of grouped images; in each vertical panel (A, B, and C), the image in the right represents the *xz* plane fluorescent image along the *D* line and corresponding to the phase-contrast image (on the *xy* plane) in the left. Also, in Fig. 4, the middle and bottom panels are representative images taken from the upper and lower parts of three different micro-colonies, each with *W_m_* labeling the transverse width of the outermost monolayer ring. The values of *W_m_* from these colonies (Fig. 4, middle and bottom figures in all panels) and others (not shown) were thus measured. The measured *W_m_* values are summarized in Fig. 5A. The average width of the monolayer areas of the micro-colonies at the 5^th^ hour was 16.6±2.2 µm (n = 19) and it enlarged slightly to 19.7±2.3 µm (n = 32) by the 6^th^ hour and to 20.0±1.7 µm at the 7^th^ h (n = 27). Therefore, these measurements consolidate the notion generated from the phase-contrast observation that the widths of the outermost monolayer rings may approach a constant value while the center cores of the micro-colonies continue to increase in size and complexity. In our cases, the *W_m_* value reached about 20 µm and stayed stably after 6–7 h of colony development. After that, the colony became too large to locate its center so that the *W_m_* values were not easy to measure. However, under the phase-contrast microscopic observation, the width *W_m_* of the outermost monolayer ring of the colonies apparently remained constant as the bacteria kept growing and the colony continued to develop. Fig. 5B illustrates collectively the developments observed above with a micro-colony during a period of 3–7 h formation.

### Bacteria at the outmost edge favoring orientations tangentially to the radial direction

**Figure 6:**
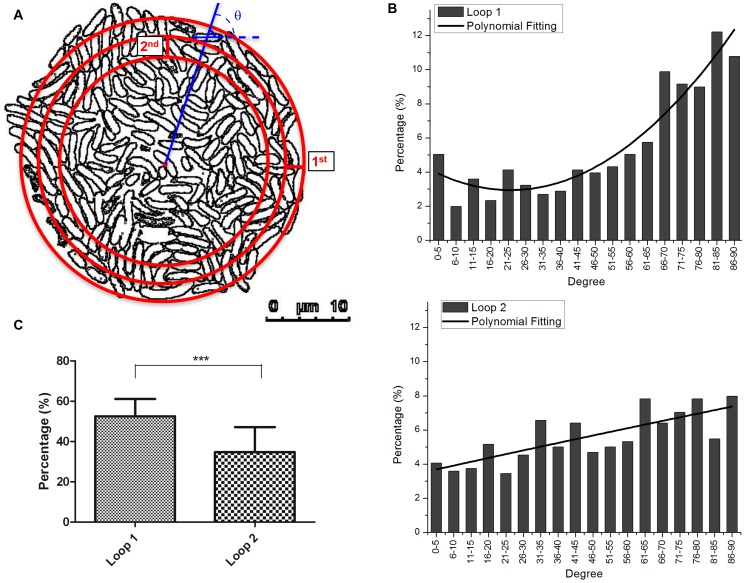
Orientation angle analysis of bacteria lying at the front edge of micro-colonies. Bacterial micro-colonies (n = 14), cultivated and imaged as shown in Fig. 2A, of roughly circular shape were used for analysis. (A) Illustration of a representative roughly circular-shaped micro-colony with the contours of individual bacteria shown. Three concentric circles were drawn to define the center of the micro-colony, the first loop that contains the outermost cells, and the second loop that is used to compare with the first loop. The two loops have the same width (2 µm). The acute angle (θ) between the cell orientation, determined from the end-to-end line segment (double-headed arrow), and the radial direction (blue line) was measured. (B) Distribution of the bacterial orientation angles. Upper panel: cells in the outermost loop 1. Order of the polynomial fitting is 2 and R square is 0.876. Lower panel: bacteria in the second loop. Order of the polynomial fitting is 2 and R square is 0.559. (C) Comparison of the percentages of bacteria in micro-colonies with orientation angles in the range of 66° to 90° between the outermost loop 1 and the one next to it (loop 2). Three asterisks mark a significant difference (*p*<0.0005 by *t*-test) when the two groups of 66°–90° were compared.

Intriguingly seen in Fig. 2A, the long axes of the bacterial rods lying at the outermost edge of the micro-colony were mostly aligned perpendicular to the radii of the colony. To consolidate this notion, the orientations of the outermost bacteria were analyzed by grouping them in two loops, each with a loop-width of approximately 2 µm (about a bacterial length) (Fig. 6A). The results obtained are shown in Fig. 6B. Most bacteria in the outermost area (loop 1) were orientated in favor of orientation angles lager than “66°” whereas those of that next to the outermost area (loop 2) showed a weaker tendency (compare the top panel with the bottom panel). Here the bacteria orientation angle “0°” denotes the orientation where bacteria orient radially, and “90°” denotes the orientation where bacteria orient tangentially, perpendicular to the radial direction. By a summation of the percentages of bacteria positioned with angles between 66° and 90° using a total of images from 14 colonies, loop 1 appeared to outnumber loop 2 significantly, with 52.5% versus 34.8% (Fig. 6C). Using computer simulations by assuming that bacterium is an elastic spherocylinder and that the elongation force, the elastic deformation and the retraction of the substratum are continuously applied to the spherocylinders, we were able to reproduce in silico a nearly circular shape of the micro-colonies with similar tangential orientations of the outermost cells (Data S1 and Figures S1,S2,S3,S4,S5), in which the outermost cells are oriented preferentially (46.3%) perpendicular (with cell orientation angle in the range of 66° to 90°) to the radial direction, compared with those located in the second loop (35.2%), in a total of 1050 simulated colonies. To account this phenomenon, our simple explanation is that the lengthening bacterial bodies and the increasing bacterial number generated outward forces that push the offspring in all directions. The most favorable orientation of the outermost rod-shape bacteria is a net result to counterbalance different interaction forces.

## Discussion

To examine the process of a single bacterium growing and dividing into three-dimensional structure of micro-colony, we used pathogen *E. coli* O157:H7 as a model for it is more motile than that of the K-12 strain. Apparently in our observations, there was no discernible difference between the two when grown on the agar surface and observed under the microscope; the bacteria slid in parallel and formed 4-cell arrays after the early two fissions. Then, the bacteria continued to divide into cell aggregates of monolayer that gradually gave a nearly circular appearance. By using bacteria expressing GFP from pQE60 and growing on LB-agar slides, we have seen fluorescence intensity variations among the bacterial cells. After the 1^st^ fission, the two daughter cells yielded a similar intensity of GFP. The equality of fluorescence intensity of the daughter cells was gradually lost over the next few generations (Fig. 2B). In one follow-up (data not shown) of the eight-daughter clusters, 89% of the aggregate counts (n = 36) gave daughter cells at apparently dissimilar levels of GFP intensity, and the intensity equality was completely lost when the 16-cell clusters were examined. Since we did not drive the GFP under a promoter inborn from the bacterial chromosome, our observation may simply represent a condition of offspring that the variation in fluorescence intensity has to do with the plasmid.

During the micro-colony development, we have seen that, at the center of the bacterial monolayer, a dense dot appeared and subsequently developed into a center core, which was discernable from the outer ring by phase-contrast microscopy. As time passed by, the center core grew larger and larger and appeared darker and darker. The dark appearance of the center core of the micro-colony was confirmed to be organized differently from monolayer by developing into multiple layers. On the other hand, the outermost ring formed dynamically and expanded outwards with continuous re-configuration of the monolayer. And the ring width increased slightly at the early phase and stayed nearly constant thereafter (Fig. 5).

We reason that the first appearance of the center core and the outer ring is due to a disparate spot arising from cells being pushed up at the center of the nearly circular monolayer. Conceptually, the pie-like bacterial lawn keeps expanding as a result of increasing individual cell volumes followed by an increase of cell number after active divisions. These volume expansion and number increase generate expanding forces in all directions. Before the dense dot formation, the summed-up expanding forces must sufficiently overcome the inward forces of friction/retraction generated by interactions among bacteria and that between the substratum and the bacterial cells.

Due to the fact that the more cells are produced, the more interacting surfaces and interaction forces are accumulated. As a result, the inward force gradually augments and finally reaches a level that is greater than the summed-up outward force of expansion. Cells in the center of bacterial lawn are then pushed up, to initiate an on-top second layer that ends up with appearance denser than the rest of monolayer cells under phase-contrast microscopy. As the colony continues to develop, a third layer of cells could be seen at the center for a similar reason, while the micro-colony keeps developing.

Our simple explanation for the fact that the widths of the outer rings increase slightly and then stay around 20 µm (Fig. 5A) during the micro-colony development is as follows. In the multilayer region, cells on the first layer are so closely packed that a new-born cell will push its neighbors and at the same time be pressed by the neighbors' reactions. Under these interactions, the cell will first deform, and then try to relax the deformation either by escaping into an upper layer or by sliding outward. The fact that the multilayer region monotonically increases its area (Figs. 3A and 5B) suggests that not all the new-born cells escape into the upper layer and those remain in the first layer succeed in sliding outwards to relax the deformation. Therefore, cells in the first layer presumably do not deform very much, so that the first layer as a whole could be regarded as an assemblage of nearly incompressible particles. This in turn suggests that while the first layer expands, its “internal pressure”, which exhibits itself as the force per unit length (represented by the red arrow shown in Fig. 5B), along the monolayer-multilayer boundary, remains roughly constant. With a constant force per unit length pushing outward and a constant (on average) frictional force per cell that counteracts this force, the number of cells per unit length that the multilayer region could push outward should remain nearly constant; this would then result in that the transverse width of the outermost ring, i.e., the width of the monolayer, remain roughly constant. In other words, this phenomenon, reflecting a balance between the cell-expansion force and the cell-substratum retraction force, may prevail in the outermost ring of monolayer.

**Figure 7:**
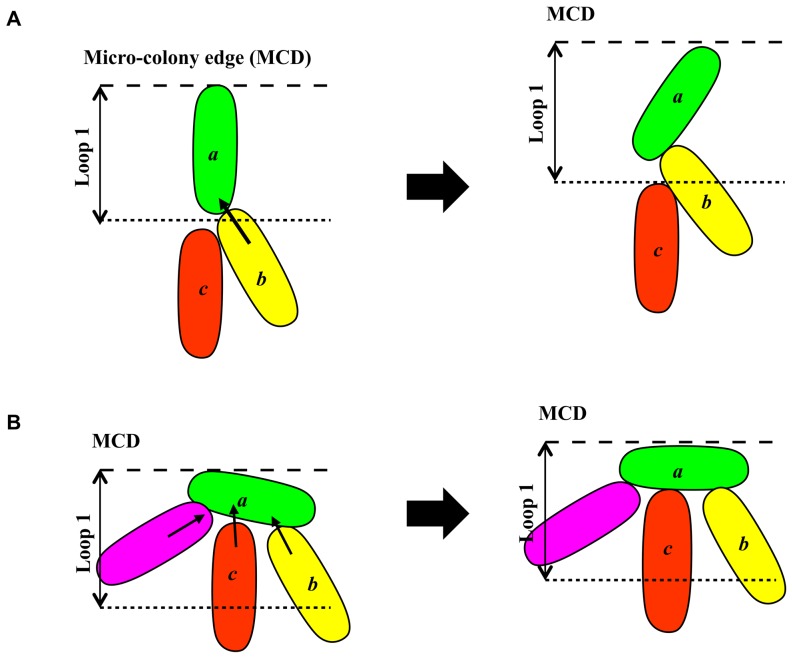
Model for formation of tangential orientation when outermost bacteria receiving a torque. (A) A likely consequence of a cell in radial orientation. Note, the limited contact surface provided by *a* and a torque exerted by *b*. (B) A tangentially positioned cell *a* before and after receiving multiple torques that cancel out each other.

Consistent with this notion is the observation that most bacteria at the front edge of micro-colony preferentially align themselves tangentially (Fig. 6). That these rod-like cells at the outermost edge favorably organize roughly in a parallel to the tangential direction could have a simple mechanical explanation. Consider cell *a* in the outermost edge (see Figs. 7A and 7B). When pushed by cell *b* in the neighboring inner ring, if *a* initially points radially outward, then *b* will leave a small room for another cell *c* nearby, to get close with, to touch and push *a*. Since *c* might provide a counter-torque that can balance the torque exerted by *b*, the condition that it is prevented from touching and pushing *a* permits *b* to freely push *a* and change *a*'s initial orientation. On the other hand, if *a* initially orients itself more or less tangentially, then even when *b* is in contact with *a*, there will still be sufficient room for *c*, and other cells as well, to come closely and interact with *a*. Thus, it is very likely that the torque exerted by *b* will be balanced by counter-torques exerted by *c* and other cells. This ensures that *a*'s initial tangential orientation remains largely unchanged. By so reasoning, as the micro-colony continues to grow, more and more cells in the outermost edge adopt an orientation that is more or less tangential to the micro-colony.

**Figure 8:**
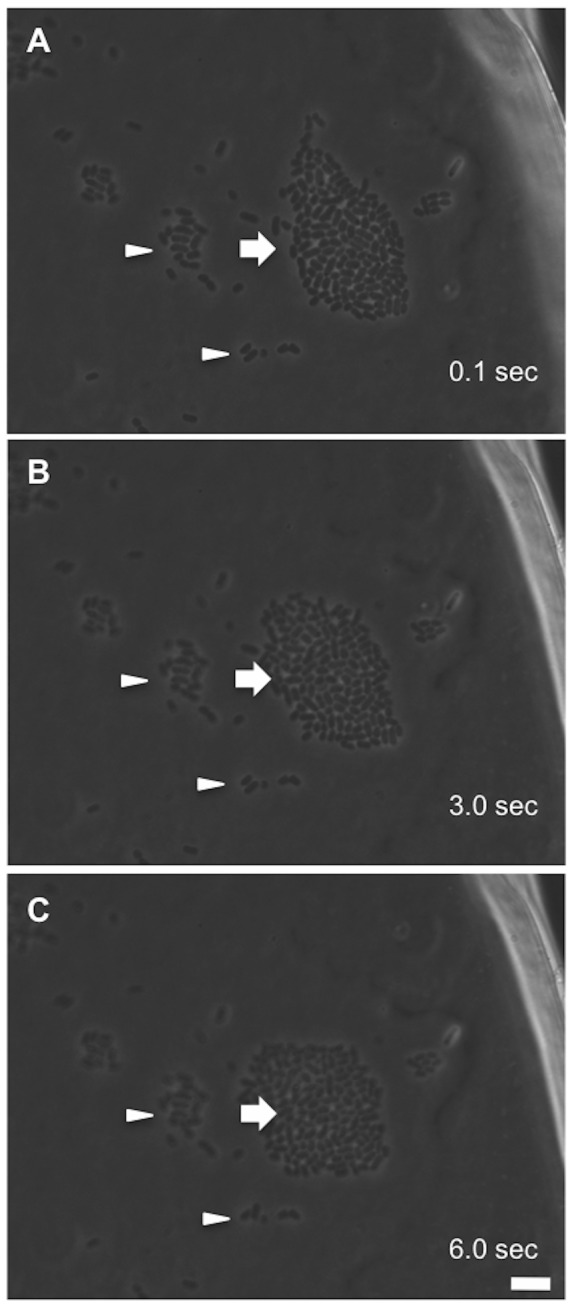
Interactions of bacteria to bacteria and bacteria to substrate surface as revealed by perturbing the formation of micro-colonies. Bacterial divisions and development into micro-colonies were carried out on a layer of thin agar immersed in LB with a coverslip-sandwiched chamber as that in Fig. 2. The chamber was lightly tapped and responses of the bacteria were immediately followed by time-lapsed microscopy. Arrowheads indicate those bacteria stayed attached to the surface but turning and vibrating could be seen with many of these cells. Arrow highlights bacteria behaving as a group that restructured their gross morphology, from a spindle-like structure (A) transiting into a near spherical shape (C), and re-positioned themselves actively (see Movie S1 in Data S1). Note: no apparent bacterial division could be seen within this short period of 6 seconds. Scale bar: 10 µm.

A boundary between the 2^nd^ layer and that of the 3^rd^ is not as clear as that between the 1^st^ layer and the 2^nd^ layer in the micro-colony growth. Neither is the boundary between the 3^rd^ and the 4^th^. This could probably be due to the fact that bacteria were frequently seen squeezing between layers near the center during the layer stacking (Figs. 3A and 3C). It is then conceivable that, under our conditions, all bacteria do actively grow including the central zone. Previously, the increase of entire radius of a growing colony has been reported to be linear with time (instead of being exponential) [21] and this has been explained by that active growth and proliferation only occur to the bacteria at the edge of the colony. As to development on solid agar, nutrient and oxygen accessibility may limit the late-stage bacterial growth in a colony. [22]. In our study, instead of following bacterial colonies growth on hard agar, we have examined single bacteria that are on an agar surface immersed in a rich medium to grow from singles to micro-colonies. By so doing, we have augmented the nutrient and oxygen accessibility and minimized the perturbation due to supplements' limitation. Our notion that colony morphology resulted from a dynamic result of force balancing could be substantiated by observing the outcomes after perturbing a micro-colony. We hypothesized that, within a few seconds after micro-colony disruption, enlargement of the bacterial cell is so small that the body elongation force could be negligible. Dispersed bacteria loosened from the micro-colony then may move as a group if bacterium-bacterium interactions remain uninterrupted. Also, since individual bacteria vary in both physiological and divisional statuses as seen from different GFP intensities, the forces attaching bacteria to the agar surface may vary individually in strength. By so reasoning, some bacteria in the micro-colony may have stronger forces toward the agar surface than the others and those bacteria are likely to stay unmoved when a light agitation occurs to the micro-colonies. Therefore, we tapped the “culture chamber” by a gentle click when micro-colonies were about to form dark centers and the imaging was resumed immediately. Figure 8 shows images of perturbed bacteria so taken for 6 seconds. As expected, we found that some bacteria did stay unmoved but body vibrations were seen with many bacteria. On the other hand, some bacteria moved as a group while staying as a monolayer (Movie S1). In this case, bacteria with a number slightly over one hundred re-organized their gross shape shortly, from a spindle to nearly a sphere. Taken together, these different pattern presentations of bacteria after micro-colony disruption strongly support our notion that constant interplay of forces does exist within a growing micro-colony. Furthermore, basing upon the fact that we have seen active dividing bacteria on all three dimensions, our results would very represent an observation of a bacterial rod growing on a nutritious surface in the process of micro-colony development.

## Supporting Information

Data S1 Simulation of a monolayer micro-colony developed from a single bacterium.
(DOCX)

Figure S1Spherocyliner as a model of *E. coli*. The length and radius of the spherocylinder are 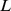 and 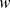, respectively. Vectors 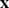 and 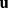 (of unit length) specify, respectively, the center and orientation of the spherocylinder. The line segment defined by 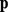 and 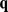, the centers of the hemispherical caps, is called the body axis of the cell.(TIF)

Figure S2Determination of the contact points. In (A) and (B), the shortest distance 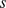 between the body axes of two interacting cells uniquely defines the contact points 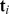 and 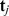, which in turn determine the direction of the cell-cell elastic interaction 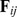. In (C), the contact points 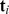 and 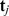 cannot be uniquely defined by 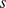, so 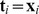 and 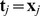 are used instead.(TIF)

Figure S3Growth curves of different maximal cell lengths. Cell length, in unit of 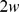, as a function of time, in unit of 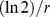. 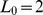 and 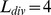. When 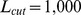, the generation time is about 1.(TIF)

Figure S4Angle analysis. To compute the distribution of cell orientation, concentric circles of radii 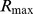, 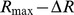, 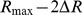, etc. are drawn, where 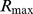 is the average distance from 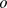 of the 10 cells lying farthest away from 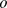. Choosing 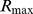 as an average over 10 farthest cells ensures that the number of cells lying in the outmost annular region will not be too small.(TIF)

Figure S5 Distribution of cell orientations from simulated micro-colonies. (A) Distribution of orientation angles for cells in the outermost loop 1. (B) Distribution of orientation angles for cells in the loop next to loop 1 (i.e., loop 2). Order of the polynomial fitting is 2 and R-square is 0.973 in (A). In (B), order for polynomial fitting is also 2 but R-square is 0.976. (C) Comparison of the percentages of bacteria with orientation angles in the range of 66°–90° between the outermost loop 1 and that of loop 2 in the simulated micro-colonies (n = 1050). Three asterisks mark a statistically significant difference between the two groups (*p*<0.0001 by *t*-test).
(TIF)

Movie S1 Continuous imaging of disrupted micro-colonies by using time-lapse microscopy. Bacterial culture and micro-colony disruption performed in Figure 8 were recorded for 6 seconds with images taken every 0.1 sec.
(AVI)
