# Supplementary material for: Bacterial Colony from Two-Dimensional Division to Three-Dimensional Development
Source: PLoS One. 2012 Nov 14;7(11):e48098. doi: 10.1371/journal.pone.0048098 (PMC3498271; doi:10.1371/journal.pone.0048098)
Supplement: Data S1 — Simulation of a monolayer micro-colony developed from a single bacterium. (DOCX) [file pone.0048098.s001.docx]

**Simulation of a monolayer micro-colony developed from a single bacterium**

To simulate the development of a bacterial micro-colony, the very first issues to address are what physics and which governing equations are involved. It is known that water is a very viscous medium for motile microorganisms [1], so the inertial effect is usually ignored when considering motile bacteria in water. For sedentary bacteria growing and dividing on LB agar, the substratum, agar, is more viscous than water whereas the surrounding medium, air, is much less viscous than water. Because the viscous agar causes strong damping, it is tempting to neglect the inertial effect as well.

However, besides viscosity, the elasticity of substratum, agar, has to be considered. In fact, the 4-cell array presumably results from the elastic retraction of the underlying agar, which had been sheared and stretched as the mother cell elongated. When the cell division ends, the mother cell and the daughter become two individual cells that can slide past one another as a result of force relaxation. Meanwhile, the breaking cell walls and contact tips inevitably introduce slight force imbalance so that the mother cell and her daughter are not in head-to-head configuration. Therefore, as the stretched agar relaxes, the mother and the daughter slide past one another, resulting in the side-by-side 2- and 4-cell configurations (Fig. 1).

In principle, we can simulate the deformation and retraction of agar. But this is not an easy task, because we must deal with not only individual cells but also a viscoelastic continuum. Alternatively, we can treat the agar as a rigid substratum and introduce artificial interactions between cells to model the elastic effect of agar. This approach allows us to focus on cells alone, thus greatly simplifies the computation. The price to pay, however, is that the inertial effect, along with some phenomenological parameters of the artificial interactions, must be included.

***Modeling bacterial cells***

Each cell of *E. coli* is modeled as an elastic spherocylinder (Fig. S1), a circular cylinder with two hemispherical caps, of length $L$ and radius $w$. For convenience, the segment of the longitudinal axis that lies between the centers of the hemispherical caps will be called the body axis of the cell. The position and orientation of a cell are specified by two vectors: the center $\mathbf{x}$ of the spherocylinder and the unit vector $\mathbf{u}$ along the body axis.

When an *E. coli* cell grows, its length $L$ increases while its radius *w* remains constant. We assume that the growth of $L$ follows the logistic equation:

$\frac{dL}{dt}=rL\left(1-\frac{L}{L_{cut}}\right)$, (1)

where *r* is the growth rate and $L_{cut}$ is the longest possible length of the cell.

To model cell division, when $L$ reaches the division size $L_{div}$, the cell will be evenly divided into two cells. Meanwhile, fluctuations in cell orientations are introduced so that the two cells will not be in head-to-head configuration.

***Equations of motion***

According to Newtonian mechanics, the equations of motion for the cell *i* are

$m_{i}\frac{d^{2}\mathbf{x}_{i}}{dt^{2}}=\mathbf{F}_{i}$ (2)

$I_{i}\frac{d\omega_{i}}{dt}=\tau_{i}$, (3)

$\frac{d\mathbf{u}_{i}}{dt}=\omega_{i}\times\mathbf{u}_{i}$, (4)

where $m_{i}$ and $I_{i}$ are the cell’s mass and moment of inertia, given by

$m_{i}=\rho\left[\frac{4}{3}\pi w^{3}+\pi w^{2}\left(L-2w\right)\right]$ (5)

$$I_{i}=\frac{1}{48}\pi\rho\left(2w\right)^{2}\left(L-2w\right)^{3}+\frac{3}{64}\pi\rho\left(2w\right)^{4}\left(L-2w\right)+$$

$\frac{1}{60}\pi\rho\left(2w\right)^{5}+\frac{1}{24}\pi\rho2w^{3}\left(L-2w\right)^{2}$ (6)

$\mathbf{F}_{i}$ and $\tau_{i}$ are the net force and torque acting on the cell, and $\omega_{i}$ is the angular velocity of the cell.

The force $\mathbf{F}_{i}$ has three components: the cell-cell elastic interaction $\mathbf{F}_{ij}$ between the cells *i* and *j*, the friction $\mathbf{F}_{cs}$ between the cell and the substratum, and the artificial force $\mathbf{F}_{s}$ that models the effect of elastic deformation and retraction of the substratum. As cells elongate and divide, they continually push against each other. We assume that the cell-cell elastic interaction exerted by the cell *j* on the cell *i* is given by

$\mathbf{F}_{ij}=k\Delta_{ij}\hat{\mathbf{n}}_{ij}$, (7)

where is a constant, $\Delta_{ij}$ is the amount of deformation, and $\hat{\mathbf{n}}_{ij}$ is a unit vector indicating the direction of the force [2]. To determine the amount of deformation $\Delta_{ij}$, we first allow the two cells to overlap and compute the shortest distance $s$ between their body axes (Fig. S2). Then the degree of overlap $2w-s$ may be used to define the amount of deformation:

$\Delta_{ij}=\max(2w-s,0)$. (8)

If $2w>s$, then $\Delta_{ij}=2w-s>0$; the cells must deform to avoid overlapping, so there is an elastic repulsive force acting on them. On the other hand, if $2w\leq s$, then $\Delta_{ij}=0$; the cells need not deform and, as expected, the force $\mathbf{F}_{ij}$ vanishes.

The points $\mathbf{t}_{i}$ and $\mathbf{t}_{j}$ between which the shortest distance $s$ is defined are called the contact points of the interacting cells (Fig. S2). The contact points define the direction of the cell-cell elastic interaction $\mathbf{F}_{ij}$:

$\hat{\mathbf{n}}_{ij}=\frac{\mathbf{t}_{i}-\mathbf{t}_{j}}{\left|\mathbf{t}_{i}-\mathbf{t}_{j}\right|}$. (9)

As usual, the friction between the cell and the substratum is assumed to be proportional to the relative velocity of the cell with respect to the substratum:

　　 $\mathbf{F}_{cs}=-b_{t}\mathbf{v}$ (10)

where is the friction coefficient for translation on the substratum. As for the artificial force $\mathbf{F}_{s}$, virtually nothing is known about it. After much effort, we still could not find a form with which the 4-cell array can be numerically reproduced. Fortunately, experimental results suggest that $\mathbf{F}_{s}$ affects cell arrangements only in the earliest stage; from the 4-cell stage onward, its effect is negligible. Therefore, we started all simulations from the 4-cell array and assumed that $\mathbf{F}_{s}=0$ throughout the entire micro-colony development.

Finally, the net torque is given by

$\tau_{i}=(\mathbf{t}_{i}-\mathbf{x}_{i})\times\mathbf{F}_{ij}-b_{r}\omega_{i}$, (11)

where is the friction coefficient for rotation on the substratum.

***Setting parameters (Table S1)***

All lengths and times were measured in terms of $2w$ and $(\ln2)/r$, respectively. Density was set equal to the density of pure water, which in turn was taken as density unit.

Because *E. coli* cells typically have an aspect ratio about 2, *i.e*., $L/(2w)\approx2$, the length $L_{o}$ of a nascent cell was chosen from a Gaussian distribution N (μ, σ^2^) centered at μ = 2 with a variance σ^2^ = 0.0025. The division length $L_{div}$ was set equal to $2L_{o}$, so that when $L=2L_{0}$ the cell divides into two cells, each having a new, and randomly chosen, length $L_{o}$. The value of cutoff length, $L_{cut}$, was adjusted (Fig. S3) so that the cell length is equal to $L_{div}$ when cell division occurs, *i.e*., $L(t=1)=L_{div}$.

Fluctuations were also introduced into cell orientation when cell division occurs, so that there was a small angle $\theta$ between the orientations of the mother and the daughter. In all simulations reported here, $\theta$ was selected from a Gaussian distribution with mean equal to 0^o^ and standard deviation equal to 1^o^.

As mentioned above, because we were unable to find an appropriate form for the artificial force $\mathbf{F}_{s}$, we were effectively simulating overdamped cell movements. Thus, the only guideline for choosing the friction coefficients, $b_{t}$ and $b_{r}$, was that there should be no discernible inertial effect. Their values, of course, depend on how strongly the interaction pushes a cell, so an arbitrary value (0.5) was first assigned to $k$, and then suitable values for the friction coefficients were determined by trial and error.

***Numerical implementation and analysis***

We used the 4^th^ order Runge-Kutta method [3] to solve Eq. (1) and the predictor-corrector method [4] to solve Eqs. (2) – (4).

The bacterial micro-colonies thus obtained were then analyzed to find the distribution of cell orientation. Drawing two concentric circles centered at $o$, the center of mass of the initial assembly, we delineated an annular region between two radii $R_{\max}$ and $R_{\max}-\Delta R$, where $R_{\max}$ is the average distance, from $o$, of the 10 cells lying farthest away from $o$ and $\Delta R$ is equal to 2. (Fig. S4). For every cell whose center $\mathbf{x}$ was located in the annular region, the acute angle between the orientation vector $\mathbf{u}$ and the radial direction was measured. Finally, a distribution of cell orientation was obtained over an ensemble of micro-colonies. (Fig. S5)

**References**

[1] E. M. Purcell, *Am. J. Phys.* **45**, 3 (1977).

[2] A. Džiugys and B. Peters, *Granular matter* **3**, 231 (2001).

[3] W. H. Press *et al., Numerical Recipes: The Art of Scientific Computing*, 3^rd^

ed.Cambridge Univ. Press, Cambridge, UK, 2007.

[4] M. P. Allen and D. J. Tildesley, *Computer Simulation of Liquids*, Oxford Univ.

Press, New York, NY, 1987.

**Table S1. Parameters used for simulations.**

| **Parameters** | **Value** | **Description** |
| --- | --- | --- |
| Parameters of cell's characteristics | | |
| $L_{0}$ | 2 | Initial length of a cell |
| $\rho$ | 1 | Cell's density |
| Growth and division parameters | | |
| $r$ | (ln2)/1800 | Growth rate |
| $L_{div}$ | N(4, 0.44) | Division length |
| $L_{cut}$ | 1000 | Cutoff length |
| $\theta$ | N(0^o^, 1^o^) | Random deviation for the orientation of the daughter cell |
| Mechanical parameters | | |
| *k* | 0.5 | Strength of cell-cell interaction |
| *b_t_* | 0.05 | Strength of cell-substratum interaction.*b_t_* and *b_r_* are translational and rotational viscous coefficients. |
| *b_r_* | 0.004 |
| Computation parameters | | |
| $\Delta t$ | 0.25 | Time step |
| *T* | When the amount of cells is about 250, the simulation is paused. | Duration of a simulation |
