# Supplementary figures and images for: Bacterial Colony from Two-Dimensional Division to Three-Dimensional Development
Source: PLoS One. 2012 Nov 14;7(11):e48098. doi: 10.1371/journal.pone.0048098 (PMC3498271; doi:10.1371/journal.pone.0048098)

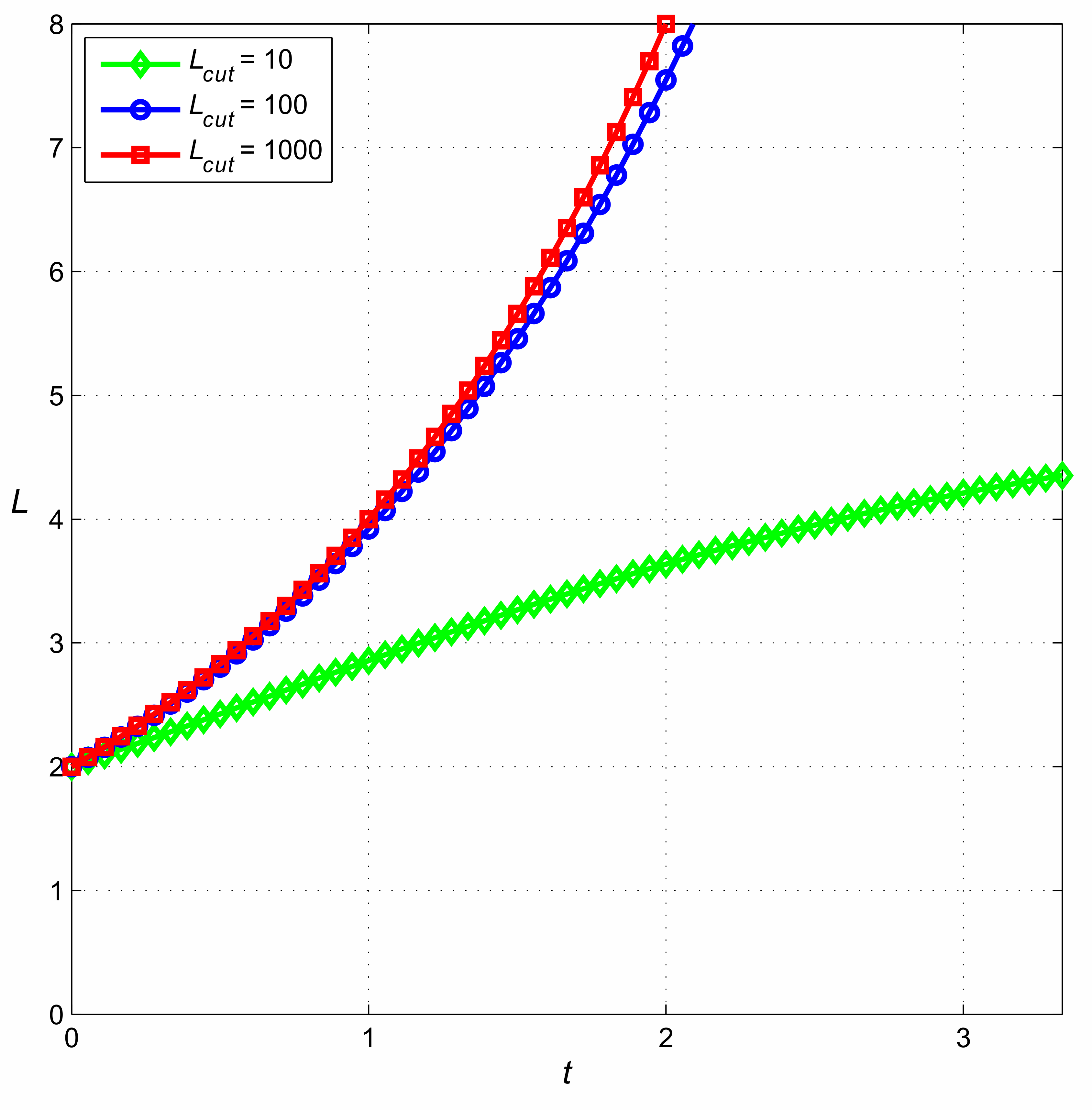

Supplement: Figure S3 — Growth curves of different maximal cell lengths. Cell length, in unit of , as a function of time, in unit of . and . When , the generation time is about 1. (TIF) [file pone.0048098.s004.tif]

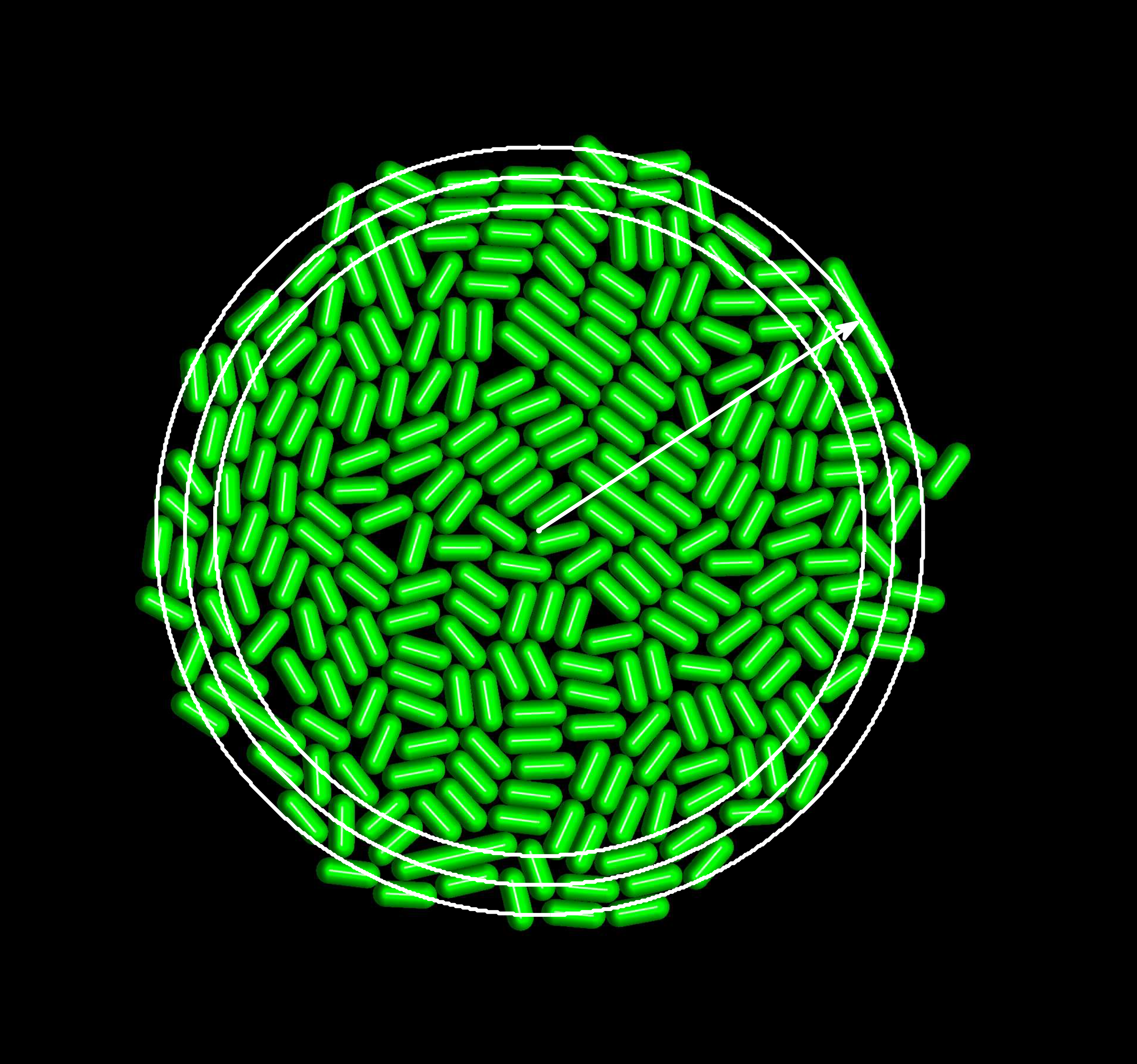

Supplement: Figure S4 — Angle analysis. To compute the distribution of cell orientation, concentric circles of radii , , , etc. are drawn, where is the average distance from of the 10 cells lying farthest away from . Choosing as an average over 10 farthest cells ensures that the number of cells lying in the outmost annular region will not be too small. (TIF) [file pone.0048098.s005.tif]

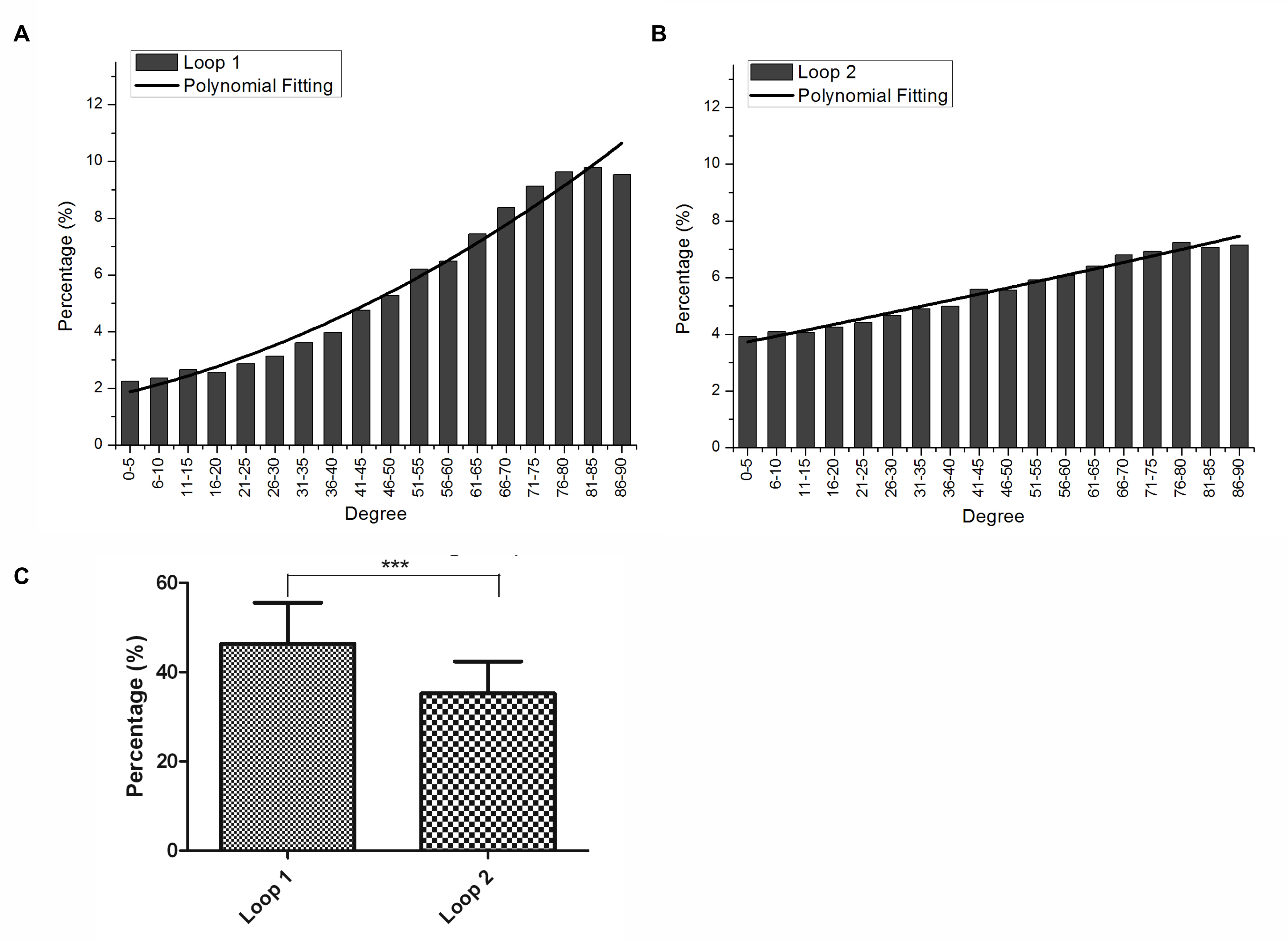

Supplement: Figure S5 — Distribution of cell orientations from simulated micro-colonies. (A) Distribution of orientation angles for cells in the outermost loop 1. (B) Distribution of orientation angles for cells in the loop next to loop 1 (i.e., loop 2). Order of the polynomial fitting is 2 and R-square is 0.973 in (A). In (B), order for polynomial fitting is also 2 but R-square is 0.976. (C) Comparison of the percentages of bacteria with orientation angles in the range of 66°–90° between the outermost loop 1 and that of loop 2 in the simulated micro-colonies (n = 1050). Three asterisks mark a statistically significant difference between the two groups (p<0.0001 by t-test). (TIF) [file pone.0048098.s006.tif]
